# Identification and characterization of a new source of adult human neural progenitors

**DOI:** 10.1038/cddis.2017.368

**Published:** 2017-08-10

**Authors:** Jinan Behnan, Biljana Stangeland, Tiziana Langella, Gaetano Finocchiaro, Giovanni Tringali, Torstein R Meling, Wayne Murrell

**Affiliations:** 1 Department of Immunology, Norwegian Center for Stem Cell Research, Institute of Clinical Medicine, Oslo University Hospital, Oslo, Norway; 2 Department of Molecular Medicine, Institute of Basic Medical Sciences, The Medical Faculty, University of Oslo, Oslo, Norway; 3 Department of Insights and Data, Capgemini, Akershus, Norway; 4 Unit of Molecular Neuro-Oncology, Neurological Institute C, Besta, Milan, Italy; 5 Department of Neurosurgery, Neurological Institute C. Besta, Milan, Italy; 6 Department of Neurosurgery, Oslo University Hospital; Oslo, Norway; 7 Vilhelm Magnus Laboratory of Neurosurgical Research, Institute for Surgical Research, Oslo University Hospital, Oslo, Norway

## Abstract

Adult neural progenitor cells (aNPCs) are a potential source for cell based therapy for neurodegenerative diseases and traumatic brain injuries. These cells have been traditionally isolated from hippocampus, subventricular zone and white matter. However, there is still a need for an easily accessible source with better yield to counter the limitations of small surgical samples of previously characterized aNPCs. Here we show that ultrasonic aspirate (UA) samples currently considered as ‘biological waste after surgery,' offer a good source for aNPCs. Furthermore, we show that culture conditions dictated the phenotype of cells across patients. The neurosphere-enriched cells were more similar to freshly isolated brain cells, while cells expanded adherently in serum conditions were similar to mesenchymal stem cells. However, cells expanded in these adherent conditions expressed some NPC and glial markers in addition to active canonical Wnt signaling. This suggests a mesenchymal-neuroectodermal hybrid nature of these cells. Finally, we show that UA-NPCs are comparable to those from neurogenic regions. Our findings suggest that UA samples can be used as a source for fresh and *in vitro* propagated aNPCs that could have various clinical applications.

An increased interest in the potential for therapeutic use of adult neural stem cells (NSCs) or neural progenitor cells (NPCs) has pushed forward efforts to find reliable sources for isolating these cells and optimizing protocols for expanding them *ex-vivo*. Several diseases are potential targets for NPC therapy, including demyelinating disorders, traumatic brain injury, stroke, Parkinson's disease, Huntington's disease, acute spinal cord injury and epilepsy.^1^ NSCs and NPCs have been isolated and characterized from neurogenic regions that were linked to neurogenesis in adult human brain such as hippocampus (HPC) and subventricular zone (SVZ).^2, 3, 4, 5, 6^ However, Ernst *et al.*^7^ showed that neurogenesis was not restricted to those regions, but occured also in regions adjacent to the neurgenic niche, such as in the striatum which is in contact with the lateral ventricle wall. Also, Nunes and colleagues isolated and characterized NPCs from a supposed ‘non-neurogenic’ region such as subcortical white matter. They sorted A2B5-positive cells and showed that NPCs constitute a part of these cells and called them white matter progenitors.^8^ Recently, Lojewski *et al.* compared NPCs from white matter (WM) to those derived from HPC and showed that the fresh primary cells isolated from tissue (annotated fresh cells) of both compartments express oligodendrocyte progenitor markers: A2B5, oligodendrocyte transcription factor 2 (OLIG2), neuron-glial antigen 2 (NG2), but not Nestin, SOX2 or CD133 which are known as NSC markers. However, neurosphere cultures established from these two compartments, WM and HPC, showed that cultured cells did express SOX2 and Nestin, but not CD133 and present very similar transcriptome profiles.^9^ Another study was able to detect the expression of SOX2 in white matter tissue (~2%) and showed that these cells are more like glial progenitors.^10^

In contrast to fetal NSCs, studies of adult NSCs/NPCs have been limited. Two culture approaches have mainly been used to enrich for these cells: one is a serum-free neurosphere culture system (EGF+bFGF/with or without PDGF),^4, 11, 12^ another is adherent serum culture with or without growth factors.^10, 13^ The known disadvantage of neurosphere culture conditions for human NSCs of being unable to grow after three passages, was countered by adherent serum culture that could generate up to 10^14^ cells from a small biopsy and followed up to 19 passages.^13^

It is important to note that both cell culturing approaches are considered established methods to enrich for NSCs/NPCs.^8, 13^ However, so far the only source for establishing such cultures from adult brain has been the small piece of tissue biopsy from patients undergoing epilepsy surgery or traumatic temporal lobe decompressions.^8^ Very few studies have used biopsy sampling from post-mortem patients,^3, 14, 15^ but these types of studies are difficult to implement due to ethical perspectives.

In this study, we investigated whether UA samples could be used as a source of NPCs. We demonstrate that UA samples, presently considered as biological waste after brain surgery, offer an abundant source for live cells that can be cultivated under different culture conditions. Based on evaluation of a wide range of protein markers expressed in fresh and culture expanded cells, we prove that UA-NPCs expanded in 10% and 1% serum express MSC and pericyte markers besides keeping high expression for some NSC/NPC markers. Protein expression together with multilineage neural and mesenchymal differentiation showed that both adherent serum cultures AD1 and AD10 resemble MSCs. The molecular profiling showed that cells isolated from fresh samples are clearly different from cells cultured in all three conditions. However, neurosphere cultures showed better similarity to fresh brain tissue than the adherent serum cultures. Comparing neurosphere cultures to serum cultures, we identified 2321 differentially expressed genes (DEGs) and several dysregulated signaling pathways such as Wnt, ECM, ribosomal proteins, axon guidance, Erk and PI-3 Kinase pathways. Finally, we show that UA-NPCs enriched under sphere conditions express comparable stemness markers to those obtained from neurogenic regions: SVZ and HPC.

## Results

### Ultrasonic aspirate samples from adult human brain contain large numbers of viable cells that can be cultivated in both serum-containing and serum-free culture conditions

Normal NSCs/NPCs from the adult human brain are notoriously difficult to obtain and propagate. In this work, we postulated that living cells from UA samples which are considered as biological waste after epileptic surgery, might provide a promising source for future stem cell therapy. To test this hypothesis, we isolated these cells from adult human brain and propagated them under different culture conditions. Samples from 14 patients that underwent epilepsy surgery of temporal lobectomy and cortical dysplasia, were used in this study. Patient diagnosis, age, and gender are shown in Supplementary Table 1. The UA samples come in sterile bags that contains fluid and fine fragments of brain tissue collected during surgery via torsional oscillation and longitudinal vibration. The irrigated saline solution containing the small tissue fragments is aspirated directly into the bag. Fresh samples were collected from the operating theatre and processed directly or within a few hours after surgery.

Single cell suspensions were prepared from these samples following the protocol developed by us for processing brain tumor tissue from UA samples.^16^ Total cell count, viability and cell yields are shown in Table 1. Cell viability in fresh samples ranged between 37-82%. The number of viable cells ranged between 10 × 10^6^ and 330 × 10^6^, and the cell yields ranged between 3.4 × 10^6^ and 14 × 10^6^ per gram of tissue; mean±S.E.M. (6.7±1.3 × 10^6^) (Table 1). The RNA integrity number of fresh cells ranged between 4.6-6.6 (mean±S.D. = 5.6±0.9), and between 7.2–9.8 (mean±S.D. = 9.2±0.8) for cultured cells.

**Figure 1:**
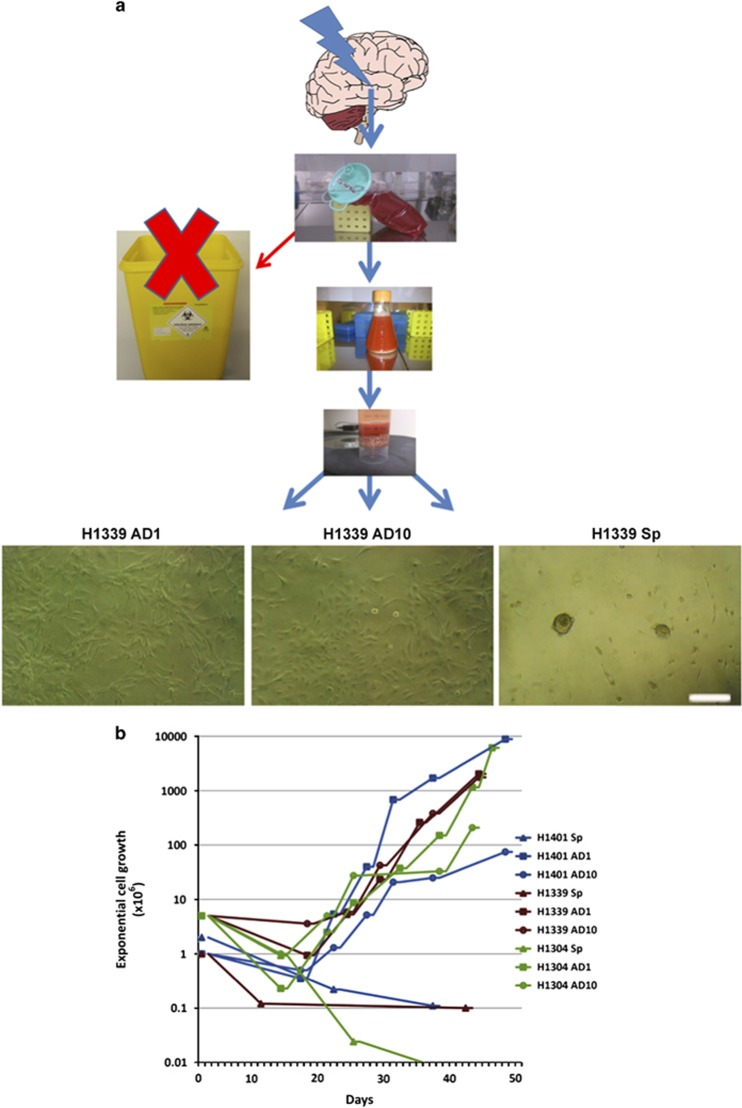
Ultrasonic aspirate (UA) samples offer a new source for cultivating adult NSCs. (**a**) UA sample was processed into single cells and cultivated under three sets of different culture conditions: adherent culture supplied with 1% FBS+TGF*α*+bFGF (AD1), adherent 10% serum culture (AD10), and neurosphere culture (Sp), serum-free culture. Scale bar, 250 *μ*m. (**b**) UA cells grow exponentially in both adherent cultures AD1 and AD10, while the number of cells decreased in Sp with passaging

To investigate the possibility of establishing primary cell cultures from UA samples, we adopted three culture protocols to cultivate these cells. Two protocols were established for expanding neural progenitors in adherent condition (1) the traditional adherent protocol in which medium is supplemented with 10% FBS serum (here after termed AD10) and (2) established by us,^13^ an adherent protocol with low serum in which medium is supplemented with 1% FBS+TGF*α*+bFGF (here after termed AD1). The third protocol is the well-known protocol of neurosphere culture that promotes floating sphere formation in serum-free conditions supplemented with basic fibroblast growth factor (bFGF) and epidermal growth factor (EGF). As reported by others for establishing neurosphere culture from neurogenic regions, UA cells formed nice floating spheres in this condition, but did not expand in cell number, and were not able to be passaged more than P3. On the other hand, cells expanded in adherent serum conditions were exponentially proliferative (Figure 1), but we used only low passages (P3-P8) in this work.

Thus, the UA samples, until now considered as biological waste after epilepsy surgery, offer an abundant source of viable cells that can be used for direct analysis or expanded under different culture conditions.

### Phenotypic characterization of fresh and culture expanded UA-NPCs by high-throughput flow cytometry analysis

**Figure 2:**
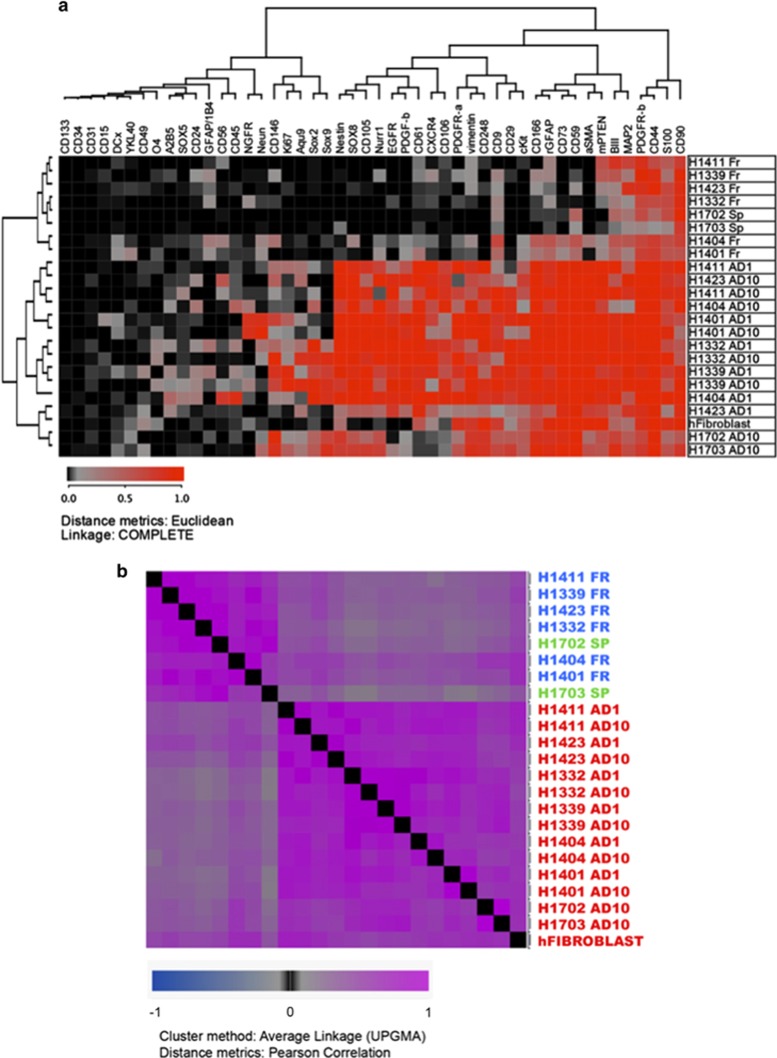
Protein expression of 48 surface and intracellular markers showing the difference in protein expression in fresh (Fr) and culture enriched cells under serum-free sphere condition (Sp) and two adherent sets of conditions, AD1 and AD10. (**a**) Hierarchical cluster analysis (HCA) of protein expression, performed by flow cytometry, showing a clear difference between fresh and culture expanded cells under serum conditions. The analysis includes expression of 48 surface and intracellular proteins in the set of 23 samples that belong to eight patients, in addition to human dermal fibroblasts. Eight patient are represented with four types of samples: fresh tissues (Fr), sphere culture (Sp) and cells cultured in 1% FBS (AD1) and 10% FBS-containing media (AD10). (**b**) HCA with distance matrix encompassing the flow cytometry analysis of fresh brain cells and cultured cells from eight patients (each represented with three samples), all samples stained with 48 markers. This analysis shows which samples are similar

To characterize and assess the protein expression level in fresh and culture expanded cells, we performed flow cytometry analysis for a wide range of surface and intracellular markers related to stemness, neural and mesenchymal lineages. The protein expression of freshly isolated cells was checked in nine patients. Fresh and cultured cells from six patients were compared side-by-side, in addition to two sphere cultures. 48 protein markers revealed a clear difference in the protein expression profile of 18 fresh and culture expanded samples (AD1 and AD10) (Supplementary Table 2). Hierarchical cluster analysis (HCA) and HCA with distance matrix showed distinct separation between the fresh tissues, sphere cultures and the adherent cell cultures (Figures 2a and b). Several glial, neural and mesenchymal markers such as (GFAP, MAP2, TUBIII, S100, PTEN, PDGFR-*β*, CD90 and CD44) were expressed in a high proportion of fresh cells and continued to be even higher in cultured cells in both AD1 and AD10 conditions. The expression of stemness and proliferation markers (Nestin, PDGFR-*α*, Vimentin, A2B5, cKit, CXCR4, CD9, SOX2, SOX8, SOX9 and Ki67) was low in fresh tissue- and neurosphere- derived cells and moderate to high in cultured adherent cells in both AD1 and AD10 conditions with some variation among patients. Other stemness and NPC markers CD133, CD15, SOX5, DCX had low expression in both fresh and cultured cells. However, the cells expanded in both adherent conditions showed high protein expression level of pericyte and MSC markers including CD248, *α*SMA, CD105, CD73, CD106, CD146, CD166 while these markers were absent or very lowly expressed in fresh tissue- and neurosphere-derived cells (Figure 2a and Supplementary Table 2). Statistically, no significant differences in marker expression were noticed between UA-AD1 and UA-AD10. On the other hand, comparing fresh cells to both tested cultured cell conditions (UA-AD1 and UA-AD10) showed significant statistical differences in 35 markers out of 49 (Mann–Whitney test, *P*-value <0.05) (Supplementary Table 2). The fresh cells of the two cases of cortical dysplasia showed higher expression of some neuroblast and stem cells markers (Supplementary Table 2). To investigate the difference between brain cells expanded in serum conditions and human fibroblasts, we checked the protein expression for the same markers on fibroblasts also. The most specific markers to distinguish brain derived NPCs with pericyte/mesenchymal properties from fibroblasts are CD105 and CD146 as surface markers and PDGF-b, Nurr1, SOX8 and SOX9 intracellularly (Supplementary Figure 1 and Supplementary Table 2). Furthermore, to test whether the cells from adherent serum condition are a heterogeneous cell population mixture of neural cells and MSC/pericyte populations, or are one cell population that expresses both properties, we transferred these cells to serum-free sphere culture conditions. These cells that were growing fast and have fibroblastoid morphology in serum conditions, stopped growing and started dying when they were transferred to serum-free neurosphere conditions. Also, these cells could not form neurospheres. The protein expression tested on two cultures showed that the transferred cells downregulated the expression of both, MSC/pericyte and NSC and neural markers, in addition to proliferation marker (Ki67), under neurosphere culture condition (Supplementary Figure 2 and Supplementary Table 3).

Thus, our data suggest a dramatic difference in the protein expression between the fresh cells derived from adult human brain and those that were expanded in serum conditions. The differentially expressed proteins showed mainly a hybrid stemness profile of NPCs and MSCs for the cells expanded under serum conditions.

### Adult hUA-NPCs have multilineage neural differentiation potential

**Figure 3:**
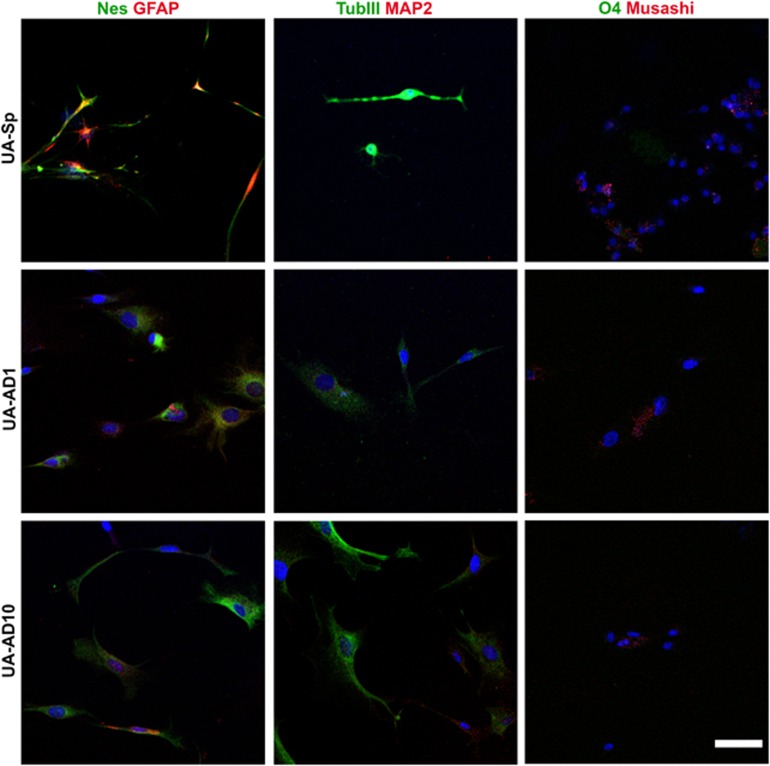
UA cells enriched in serum-free medium (UA-Sp) and serum-containing medium (UA-AD1 and UA-AD10) showed multilineage neural differentiation potential. Immunofluorescent staining of neural lineage markers (*β*TubIII and MAP2 for mature neurons, GFAP for astrocytes, O4 for oligodendrocytes, Nestin and Musashi for immature cells/progenitors) after 3 weeks of *in vitro* differentiation. We repeated this experiment on four patients; the same laser intensity and confocal settings were used to compare different culture conditions from the same patient. UA-Sp expressed the strongest *β*TubIII level. Cells from the three conditions expressed the immature markers, Nestin and Musashi. Scale bar, 20 *μ*m

To investigate the multilineage neural differentiation properties of cells derived from UA samples under different culture conditions, we subjected them to a neural differentiation protocol for 2 weeks as previously described.^16^ Under neural differentiation conditions, both neurosphere culture (UA-Sp) and adherent serum cultures (UA-AD1 and UA-AD10) gave rise to cells that express the glial marker GFAP and mature neuron marker TUBIII. Cells from UA-AD1 and UA-AD10 showed low expression for MAP2. The oligodendrocyte marker O4 was not detected in any culture conditions. However, the differentiated cells from all three culture conditions retained the expression of the NSC marker (Nestin) and the immature neural marker (Musashi) (Figure 3). This experiment was performed on cells from four patients. Marker expression in different culture conditions from the same patient was examined by using the same laser intensity and confocal settings. Thus hUA-NPCs expanded under different culture conditions showed glial and neuronal differentiation potential without completely losing the expression of progenitor markers.

### Cells expanded in serum conditions showed some mesenchymal differentiation properties

**Figure 4:**
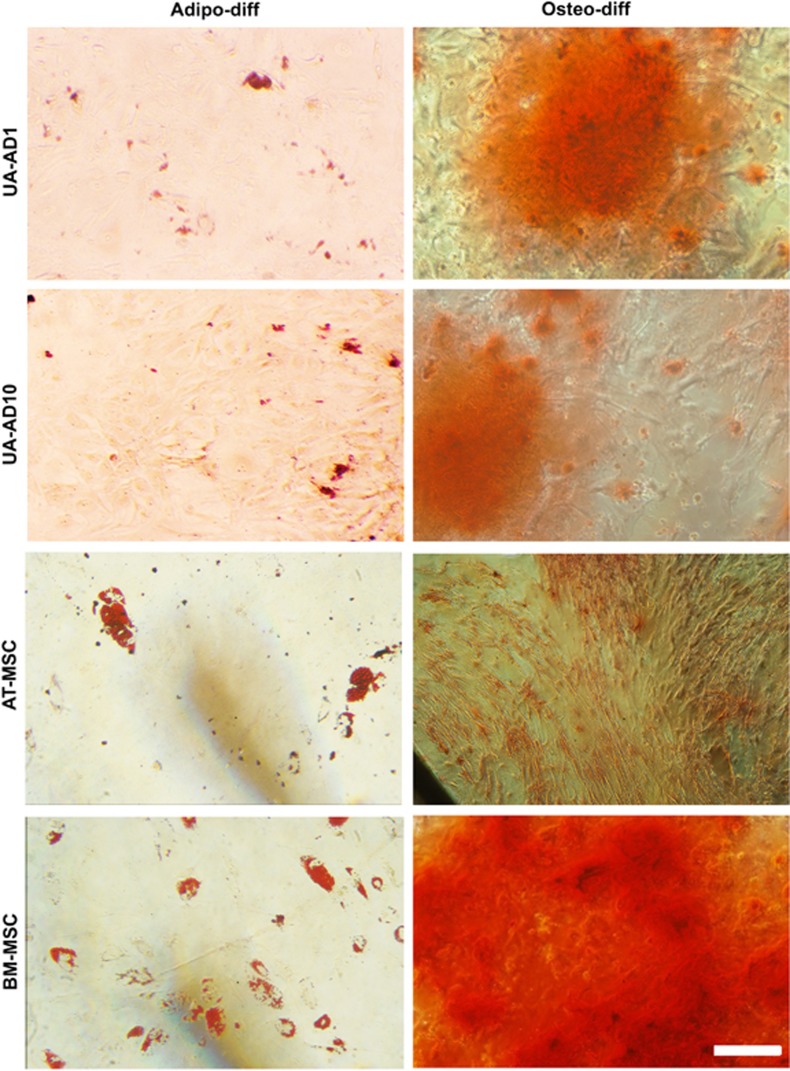
UA cells cultured in serum-containing medium (UA-AD1 and UA-AD10) showed some MSC differentiation properties. The first column shows the differentiation of UA-AD1 and UA-AD10 into adipocytes after two weeks. Oil red staining for lipid droplets showed that both UA-AD1 and UA-AD10 have accumulatedlipid droplets. However, the number of differentiated cells and the load of lipid droplets looked less than those from BM-MSCs and AT-MSCs. The second column shows the osteogenic differentiation potential of UA-AD1 and UA-AD10 after 2 weeks. These cultures showed an osteogenic differentiation property as indicated by alizarin red staining of calcium deposits. Scale bar, 250 *μ*m

To test whether the cells that are expanded in both serum-containing conditions (UA-AD1 and UA-AD10) and express MSC markers have the potential to differentiate into mesenchymal lineages, we subjected them to three lineage differentiation protocols: Osteogenic differentiation visualized by alizarin red staining for calcium deposits, and adipogenic differentiation shown by Oil-Red staining of lipid droplets, were approximately similar for both AD1 and AD10, but less than other MSCs derived from bone marrow (BM-MSCs) and adipose tissue (AT-MSCs) (Figure 4). This was done on three patients. Chondrogenic differentiation was difficult to process because of getting very small fragile cell pellets.

Thus, both cell populations UA-AD1 and UA-AD10 showed some mesenchymal differentiation properties

### Global mRNA profiling of UA samples from adult human brain tissues and ahNPCs expanded under different culture conditions show a number of differentially expressed genes and signaling pathways

**Figure 5:**
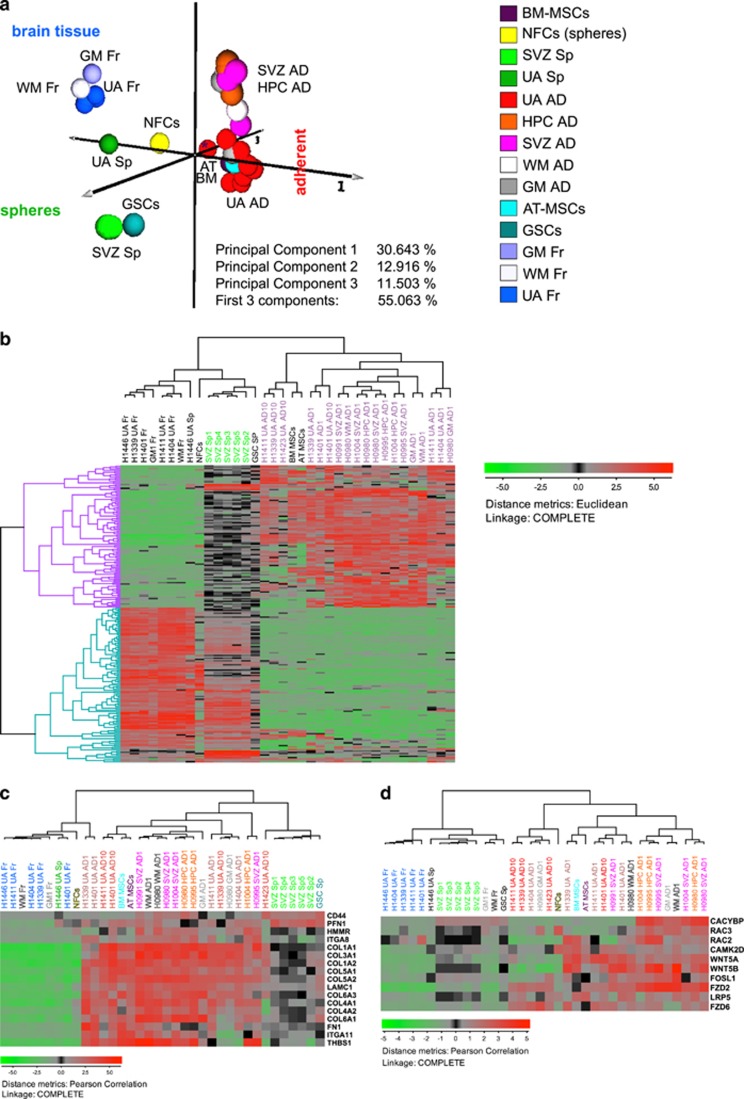
Global mRNA profiling showing the difference between fresh cells from UA samples of adult human brain and those enriched under different culture conditions. Patient samples were grouped in response to culture conditions showing some difference between UA samples and those from SVZ and hippocampus (HPC). (**a**) Global PCA analysis**.** To visualize similarities or dissimilarities between samples, we used global principal component analysis (PCA) of gene expression. Each individual sample is represented with a ball of a certain color. Principal components PC1, PC2 and PC3 were represented as axes 1–3. While the PC1 separated fresh tissues (up to the left) from neurospheres (down to the left) and adherent cell cultures incubated in serum-containing medium (right), the second PC stratified the cluster of adherent cell cultures. PC2 thus roughly separated UA samples (bottom) from the adherent cell cultures originating from neurogenic regions of the human brain such as SVZ, HPC, WM and GM. The adipose tissue MSCs and bone marrow MSCs clustered together with the serum-grown UA cell cultures. Meanwhile the sphere cultures expanded from the SVZ, SVZ-Sp, (lighter shades of green) clustered tightly together (and in the proximity of a GSC sample), the sphere culture expanded from UA (UA-Sp) (darker shade of green) clustered closer to fresh tissues (Fr). (**b**) Spheres and adherent cultures express different sets of genes**.** HCA and stringent filtering identified 224 differentially expressed genes (DEGs) with the most variable expression between the groups of spheres (SVZ-Sp) and the serum-grown adherent cell cultures (such as UA-AD, SVZ-AD, GM-AD, WM-AD and HPC-AD). These two groups are specified with the green and darker shade of orchid dendrogram colors respectively (upper panel). Dendrogram branches on the left represent genes upregulated (lilac) and downregulated in the adherent cultures (turquoise). Low expression is shown with green color while high expression is shown in red. The expression values were log_2_ transformed. (**c** and **d**) Pathway analysis. Several biochemical signaling pathways such as ECM-receptor interaction (**c**) and Wnt signaling pathways (**d**) are differentially expressed in spheres and adherent cell cultures. These pathways were identified using functional annotation analysis (https://david.ncifcrf.gov). In (**c** and **d**) are shown that both pathways are upregulated in the adherent cells. It is especially interesting that the canonical Wnt signaling that is traditionally associated with cancers (such as GBM) and brain development is active in the normal brain cell cultures

To compare the molecular profiles of UA cells, both fresh and cultured, to each other and to those derived from the neurogenic and non-neurogenic regions of the human brain (SVZ, HPC, GM and WM), we used gene expression analysis. To harness the full strength of global gene expressional profiling, we utilized principal component analysis (PCA), a method that allows identification of clusters with similar variations. PCA does not only enable an assembly of individual samples into groups and constellations but also facilitates an easy detection of outliers. Each sample is represented by a globule of a specific color. In this study, PCA showed clear separation of the globules representing tissues samples (fresh) and those representing *in vitro* expanded cells (Figure 5a and Supplementary Figures 3a-c). The constellation to the left, consisted of the globules representing fresh tissues (up to the left) and the sphere cultures (down to the left), while the oblong constellation to the right, consisted of globules representing adherent cell populations from UA and neurogenic regions (to the right, represented in different shades of red) (Figure 5a). Both UA-AD1 and UA-AD10 could be separated from those derived from neurogenic regions that were closer to WM and GM progenitors (Figure 5a and Supplementary Figure 3). Interestingly, the sphere culture from UA (UA-Sp) clustered much closer to the fresh tissues (to the left), while the five samples of spheres derived from SVZ formed a tight cluster grouping with one another (Figure 5a).

Hierarchical cluster analysis performed on 3833 filtered genes confirmed this separation too (Supplementary Figure 4). All adherent cultures expanded in the serum-containing medium clustered together in one super-branch together with BM-MSCs and AT-MSCs (represented by the red dendrogram color), while fresh and sphere cultures from both SVZ and UA in addition to neural fetal cells (NFCs) formed another super-branch represented by the green (SVZ and NFCs) and blue (fresh) dendrogram color. However, there was clear separation between AD1 cells originating from neurogenic regions and those from UA (Supplementary Figure 4).

We further compared brain cells isolated from SVZ and cultured as neurospheres, (serum-free and mitogen-containing medium, GSE31262) (group a) to the ultrasound aspirates (UA) and brain cells isolated from HPC, SVZ, GM and WM) (group b). To identify differentially regulated genes between the two groups, we used fold-change analysis. This analysis yielded 2321 differentially expressed genes (DEGs) (Supplementary File 1). To pinpoint the biochemical pathways that were differentially regulated in spheres and adherent cells we identified 1440 DEGs with the most variable expression using a filtering function (Supplementary Figure 5) and used these for the functional annotation (Supplementary File 1). This analysis identified the following pathways as differentially regulated: expression of ribosomal proteins (*P*=3.8E−35), axon guidance (*P*=3.4E−2) and the biochemical signaling pathways such as Wnt (*P*=5.9E−2), Erk & PI-3 Kinase (*P*=9.4E−3), ECM-receptor interaction (*P*=1.5E−5) and several others (Figures 5c and d,Supplementary Figure 6 and Supplementary File 1). The first two pathways were significantly upregulated in sphere cultures, while the other three were upregulated in adherent cultures grown in serum. This analysis was performed through utilizing the open-source functional annotation tool DAVID (Supplementary File 1 and Supplementary Figure 6).

To further visualize the differences between sphere cultures and the adherent cell cultures we identified 224 most variably expressed DEGs (out of the 1440 DEGs) using even more stringent filtering (Figure 5b). The hierarchical cluster analysis (HCA) of the 224 genes identified 107 genes (lilac dendrogram branches to the left) that were upregulated and 117 (turquois dendrogram branches to the left) genes downregulated in adherent cells respectively (Supplementary File 1). Comparing the five UA fresh samples to the nine cultures derived from these cells, 2385 genes were differentially expressed (Supplementary Figure 7).

The abovementioned analyses together indicate that materials harvested from UA samples enrich for cells that have molecular profiles different from those of fresh tissues. There are also strict differences between the cell cultures propagated under different culture conditions. Both UA-AD1 and UA-AD10 were slightly different from SVZ and hippocampal AD cultures and showed some similarities with MSCs. Interestingly, adherent cells showed both, an upregulated ECM pathway, and increased Wnt-signaling, the latter typically associated with embryonic development and maintenance of NSCs.

### Adult hUA-NPCs are comparable to those derived from SVZ and hippocampus

**Figure 6:**
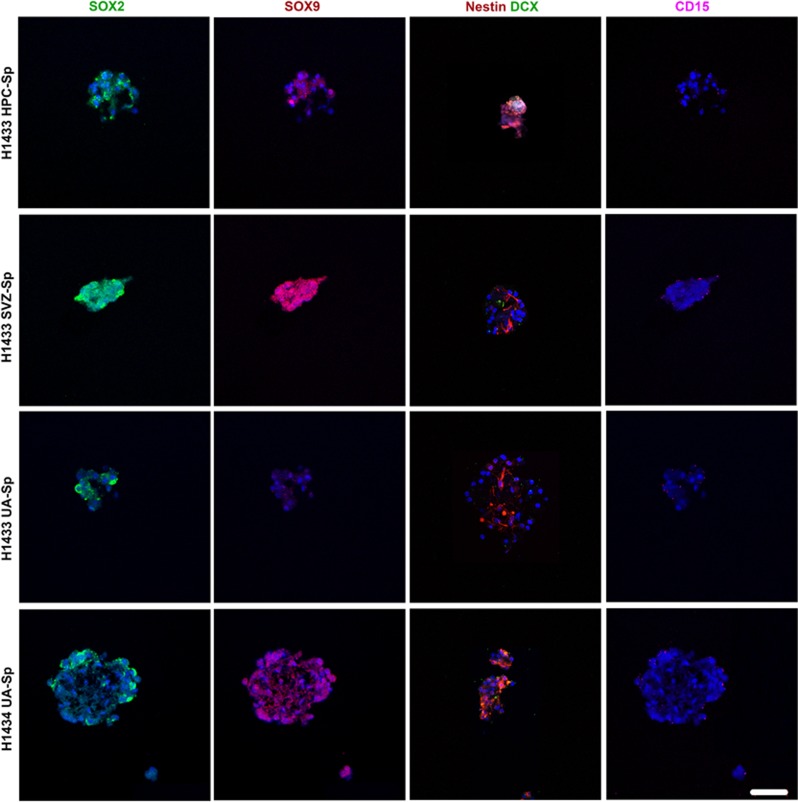
UA Cells grown in neurosphere culture express NSC markers similar to those from SVZ and hippocampus (HPC), but SVZ seems to have higher expression of SOX2 and SOX9. Immunofluorescent staining for selected NSC markers (SOX2 (green), SOX9 (red), Nestin (red), DCX (green), CD15 (purple)). We had spheres from SVZ and HPC from only one patient out of three that have UA-Sp. The same laser intensity and confocal settings were used to compare the marker expression in sphere cultures from different compartments. Scale bar, 50 *μ*m

To compare hUA-NPCs to SVZ-NPCs and HPC-NPCs and because of the limited availability of materials from these two neurogenic regions, we picked some NSC-related markers to be tested. SOX2 and SOX9 are transcription factors of the high mobility group (HMG) of the SOX family, which have been reported to be markers of proliferating NPCs and of the developing central nervous system.^17^ CD15 has been reported to be expressed by neuroepithelial cells.^18^ Nestin is a neurofilament protein and supposed NSC marker.^19^ We examined the cell expression of SOX2, SOX9, Nestin, DCX and CD15 in neurosphere sections from SVZ, HPC and UA samples. Cells from UA samples as well as from SVZ and HPC expressed SOX2, SOX9, Nestin, DCX, CD15, but SVZ seems to have the strongest expression of SOX2 and SOX9 (Figure 6). The same laser intensity and confocal settings were used in the comparison.

## Discussion

The limited amount of tissue and cells from adult human brain has been the main reason for not having proper analysis and comparison side-by-side for fresh and culture expanded cells. This in its turn made it very difficult to use ahNSCs in therapeutic settings in clinical trials. In this work, we showed that UA samples, previously discarded as biological waste following epileptic surgery, represent a valuable source, of viable cells from adult human brain to be used for future stem cell therapy ad diagnosis of multiple neurological diseases. The isolated cells were able to grow *in vitro* under different culture conditions including AD1, AD10 and neurosphere conditions. The large number of cells obtained from UA samples enabled protein and gene expression comparisons. Our work describes, for the first time, the protein and gene expression profiles that delineate the transition of fresh cells undergoing *in vitro* cultivation. Using several NSC markers, we showed that neurosphere cultured cells from UA are comparable to those from neurogenic regions.

Using UA samples as a source for extracting live cells has not been very common, mostly because it was thought that the sonication process would kill all harvested cells. This abundant source of viable fresh cells can be utilized to develop better diagnostic criteria for patients that suffer from many developmental and neurological disorders. The assessment of protein expression of a wide range of markers showed that the two patients with cortical dysplasia, included in this study, express higher percentages of several neuroblast and stem cell markers including DCX, NeuN, Nurr1, CD56, CD166, CD9, CD73, CD59, CD61 and cKit. This observation supports two previous studies that investigated the expression of a few stem cell markers (CD133, SOX2, Oct-4, c-Myc, FOXG1, KLF4, Nanog and SOX3) in cortical dysplasia.^20, 21^

Although it has been shown that the neurogenesis mainly occurs in neurogenic regions of the adult human brain including hippocampus and SVZ,^2, 3, 4, 5, 6^ proliferative progenitors have been identified and isolated from different non-neurogenic regions of adult human brain including white matter, striatum and cortex.^7, 8, 9, 10, 11^ These studies offered good support for the hypothesis of cultivation of NSCs/NPCs from UA samples. The UA samples that we introduced here as a new source for adult normal cells are mainly mixtures of white and gray matter and might contain fragments of neurogenic regions as the ultrasonic aspirator device is used for the smooth fine removal of tissue and the cleaning of the surgery site. However, the disadvantage of using these samples is that they do not have clear histological structure. On the other hand, these samples might cover the cell heterogeneity of adult brain better than a single biopsy does. The heterogeneity captured in UA samples can be noticed from the molecular profiling where the UA-Sp sample clusters closer to fresh tissue than SVZ-Sp samples. The cell heterogeneity together with the high cell yield of UA samples could offer a better source for studies aiming to sort different types of brain cells from fresh tissue for testing drugs and toxicity, and even might be a better starting material for protocols aiming to cultivate various types of human brain cells.

We cultivated cells from UA under three different culture conditions. The cells from two adherent cell cultures supplied with serum (AD1 and AD10) were growing exponentially. Under the third set of conditions, the neurosphere conditions, cells did form nice floating spheres, were able to be passaged up to P3, and expressed NSC markers, but the number of cells was decreasing with passaging. The heterogeneity of cells in this sample, and the culture system selection pressure which allows only a small population of the cells to survive could be the main reason to constantly lose cells. The fresh samples contain a lot of mature astrocytes, neurons and some hematopoietic cells which probably cannot survive under neurosphere conditions. However, although the neurosphere system is considered the gold standard for cultivating NSCs, we and others have shown that adult human brain NSCs from neurogenic and non-neurogenic regions could not be maintained more than P2-P3, and no one has shown that the number of cells is increased under this condition.^8, 11, 13^

The protocol for culturing cells in AD1 condition ('fail-safe' protocol) was established by us recently. We have shown that these cells are highly proliferative under this condition; we followed them up to P20.^13^ Our pathway analysis indicates that adherent cells exhibit increased canonical Wnt signaling, a pathway predominantly associated with cancer and embryonic brain development and considered to be critical for the maintenance and proliferation of NSCs.^22^ Previous studies showed that Wnt signaling stimulates proliferation and suppresses differentiation of several types of stem cells including NSCs and glioblastoma stem cells (GSCs).^23, 24^ This pathway was linked to the increased proliferation of GSCs recently.^25^ Although these adherent cells were not cancerous they exhibited expressional profiles indicating higher growth rates and higher renewal potential than the other samples. Most importantly, we have confirmed the safety profile of these cells and showed that those cells have normal karyotype and did not form any tumors upon *in vivo* transplantation.^13^ UA-AD1 cultures did not differ significantly from UA-AD10 in their protein expression. UA-AD1 and UA-AD10 express high levels of markers, reported to characterize perivascular MSCs/pericytes from adult human brain derived from SVZ and cortex such as CD73, CD90, CD105, *α*SMA, PDGFR-*α*, PDGFR-*β*, CD106, CD166, CD29 and Nestin.^26^ The properties of adherent cells to differentiate into some mesenchymal and neural lineages, in addition to the co-expression of MSC and NPC markers, support the hypothesis of neuroectoderm origin of the brain pericytes that was suggested by others.^27, 28^ Also, we could distinguish the adherent serum cultures from fibroblasts by the expression of CD105, CD146, Nurr1, SOX8 and SOX9. CD105 and CD146 that were absent in skin fibroblasts, and have been previously reported to characterize perivascular progenitors from different tissue, including human brain and glioblastoma.^29, 30^ Nurr1 was found to be essential for the differentiation of mesencephalic dopaminergic precursors into full dopaminergic neurons, while SOX8 and SOX9 were reported to have a role in neural crest development and maintenance of NSCs.^19, 31, 32, 33^ However, serum-cultured cells were not able to grow in serum-free culture conditions supplied with EGF and bFGF and could not form neurospheres, which means those are not the same NSCs/NPCs that grow in sphere conditions. Although the UA-AD1 cells are more similar to brain pericyte cells than NSCs, the fact that we can get up to 10^18^ cells from a small piece of sample, suggests them as potential candidates for future clinical therapy in regenerative medicine.^13^ We are trying to develop an efficient dopaminergic differentiation protocol for these cells. Currently, we believe the best use of AD1-enriched cells will be in the reprogramming field. Karow *et al.*^34^ showed that overexpressing of only two transcription factors, SOX2 and MASH1, in brain pericytes is enough to successfully reprogram these cells into fully mature neurons. Also, for a clinical application, although the AD1 conditions use low percentage fetal bovine serum, the option to replace it with autologous serum or to use commercially chemically defined animal and human serum-free culture media approved by FDA seems more appropriate.

In summary, we have shown for the first time that UA samples offer a great source for obtaining adult human brain cells. UA fresh cells have good viability and could be cultivated under different culture conditions. Importantly, we showed the differences in protein and gene expression between fresh cells and their *in vitro* enriched progenitors. Although the culture conditions were the main dictating factor for the type of cells enriched under each set of conditions, small differences were noticed between cultured UA cells and those expanded under the same conditions from neurogenic regions. However, the analysis of their molecular profile indicates that the UA neurosphere cultures are more similar to fresh brain cells, while across the patients, cells from both adherent conditions AD1 and AD10, showed similarity to each other and expressed MSC/pericyte markers. However, the co-expression of MSC/peritcyte markers and neural and glial progenitor markers, in addition to active canonical Wnt signaling in the adherent cells, suggests an apparently hybrid phenotype. Some kind of dual mesenchymal-ectodermal potency of these cells is suggested and might explain less successful reprogramming efforts to get mature neurons from these cells.

Anyhow, we believe that UA samples offer a valuable source of cells that could be used in future diagnosis and stem cell therapy.

## Materials and methods

### Patient samples collection

UA samples were acquired from 12 patients that underwent surgery for refractory epilepsy. Informed consent was obtained from all patients before getting the samples. Experimental procedures were carried out in accordance with the guidelines of the Norwegian National Committee for Medical Research Ethics after being approved by the Regional Ethical Committee (REC South-East S-07321d) and by Best Ethical Comittee, Milan, Italy. The histology confirmation of not developing tumor was confirmed after operation and two years later. The tissue fragments within saline solution were obtained by ultrasonic aspiration with a Sonoca 300 ultrasonic dissector/aspirator (Söring, Quickborn, Germany) during surgery and aspirated directly into a sterile plastic bag. After the surgery was finished, the sterile bag was closed and transferred to the laboratory.

### Establishing primary cell culture

Fresh UA samples were processed directly or within a few hours of the surgery. All the liquid fraction with tissue fragments were spun down, washed and then weighed. The collected tissue sample was divided into three fractions: one was cryopreserved in FBS/DMSO (BioChrome, VWR and Sigma, St Louis, MO, USA), one was snap frozen for protein and RNA extraction, and one was weighed and then processed into a single cell suspension following the protocol established by us for processing UA samples from brain tumor patients.^16^ Cells were counted and plated in three different sets of culture conditions: (1) adherent culture expanded in 'failsafe' medium (AD1): DMEM/F12-GlutaMAX medium supplemented with 1% FBS, 2% B-27 (Gibco, Life Technologies, Paisley, UK), 10 ng/ml basic fibroblast growth factor, 20 ng/ml TGF*α*, 10 mm HEPES (Lonza, BioWhittaker, Cologne, Germany), 100 U/ml penicillin/streptomycin of both (LonzaBioWhittaker) and 2.5 *μ*g/ml heparin (Leo Pharma AS, Esbjerg, Denmark);^13^ (2) traditional adherent serum culture expanded in DMEM/F12-GlutaMAX medium, 10% FBS, 10 mm HEPES, and 100 U/ml Penicillin/Streptomycin. (3) neurosphere culture conditions which is serum-free sphere culture containing DMEM/F12-GlutaMAX medium, 10 ng/ml bFGF, 20 ng/ml EGF (both R&D Inc, Minneapolis, MN, USA), 1:50 B27-supplement, 100 U/ml penicillin/streptomycin, 1 ng/ml Heparin (Leo Pharma), and HEPES 5 mM. Cells were fed with EGF and bFGF twice a week and cell culture medium was refreshed on day 7. Adherent cultures were passaged upon reaching 70–80% confluence, while neurosphere cultures were passaged once a month. A human dermal fibroblast cell line (NHDF) was obtained from Lonza and cultured according to the protocol CC-2511 (Lonza).

### Flow cytometry

UA cells from fresh samples that were processed into single cells without being cultivated and cultured cells from both different adherent conditions (AD1 and AD10) were first washed with FACS buffer (PBS containing 4% FBS). Surface and intracellular staining antibodies are shown in (Supplementary Method Tables). Intracellular staining was performed by using BD Cytofix/Cytoperm Kit (Torreyana Rd, San Diego, CA, USA). Cells were first fixed for 30 min, washed and permeabilized, and then incubated overnight with primary antibodies. After washing the cells, matched secondary antibodies were added and incubated for 2 h. Cells during all incubations were kept at +4 °C. Cells were then washed and analyzed by flow cytometry, using an LSR Fortessa cell analyzer (BD Bioscience, San Jose, CA, USA). 10 000 events were counted at least and FlowJo software was used for final data analysis (Tree Star, Ashland, OR, USA).

### Multilineage neural differentiation

The cover slips (Thermo Scientific, Brunswick, Germany) were placed in 24-well plates and coated with poly-L-ornithine (28 *μ*g/cm^2^, 0.1 mg/ml; Sigma-Aldrich) plus laminin for 48 h at 37 °C. Cover slips were washed twice and cells plated in differentiation medium consisting of DMEM/F12-GlutaMAX medium supplemented with 4% FBS, pencillin/streptomycin, HEPES, B27-supplemented with retinoic acid (Gibco, Life Technologies).

### Multilineage mesenchymal differentiation assays

#### Osteoblastic differentiation

Cells from P3-P7 were plated in triplicate at 10 000 cells/well in 24-well plates. Cells were allowed to grow in osteogenic differentiation medium (Grand Island, NY, USA) for 2 weeks. BM- and AT-MSCs were used as controls. Differentiated cells were rinsed carefully with PBS and fixed with ice-cold 70% ethanol for 1 h. Cells were carefully rinsed three times with water and stained for 10 min with Alizarin Red and then washed carefully four times. Undifferentiated cells were used as a negative control, and staining of calcium deposition was visualized via light microscopy.

#### Adipogenic differentiation

Cells were plated in triplicate at 20 000/well in 24-well plates, allowed to reach 100% confluence, and then the original expansion medium was replaced with adipogenic differentiation medium as previously reported.^30^ BM- and AT-MSCs were used as positive controls, and undifferentiated cells in original growth medium were used as negative controls. The induction of adipocytes was assessed after 2 weeks using oil red O stain (Sigma-Aldrich) as an indicator of intracellular lipid accumulation.

#### Chondrogenic differentiation

Chondrogenic induction was done by pelleting cultured cells (5 × 10^5^ cells per pellet) and then by putting them through the process of preparing an alginate scaffold according to an established protocol.^35^

In all three mesenchymal differentiation assays, the medium was replaced twice a week.

### Neurosphere sectioning

Spheres were spun down at 300 g for 5 min, the supernatant was removed and 0.5 ml of fixation buffer of the BD Cytofix/Cytoperm Kit was added directly (Torreyana Rd, San Diego, CA, USA). After 24 h, spheres were spun down, fixative buffer was discarded and 0.5 ml of 20% sucrose solution was added for another 24 h for cryoprotection. After this, spheres were resuspended in 50 *μ*l O.C.T and then embedded in a specimen block which was filled with O.C.T (200–300 *μ*l) and previously placed on dry ice for 30 s. After complete freezing, blocks were transferred to −20 °C. Sphere sections of 14 micrometer thickness were prepared by cryostat and kept at −20 °C for later use.

### Immunofluorescent staining

For immunocytochemstry, cover slips with the fixed cells attached were incubated with the indicated primary antibodies overnight, washed 2 × 2 min in PBS followed by 1 × 2 min in TBST and subsequently incubated with the appropriate secondary antibodies for 2 h at room temperature. After this, cover slips were gently washed 2 × 2 min in PBS, then stained for 10 min in Hoechst (1:1000, Sigma Aldrich), washed once with PBS and once with dH2O to clean all slat-crystals and mounted with Prolong Gold anti-fade reagent (Invitrogen, Eugene, OR, USA).

For immunohistochemistry staining of neurosphere sections, frozen sections were allowed to be defrosted for 15 min, rehydrated 2 × 5 min in PBS, and then 2 × 5 min in Tris-Buffered Saline Tween-20 (TBST) and incubated with blocking buffer (5% milk powder in TBST and 10% goat serum) for 30 min at room temperature. The primary antibodies, monoclonal mouse anti-human SOX2, rabbit anti-SOX9, rabbit anti-SOX8, goat anti-CD15 and human-specific anti-Nestin, were used for this staining. Slides were incubated overnight at 4 °C, washed 2 × 5 min in PBS followed by 2 × 5 min in TBST, and then incubated with secondary fluorescent antibodies conjugated to Alexa Fluor 488, Alexa Fluor 594, Alexa Fluor 647 (1:500), incubated overnight at 4 °C, washed 3 × 5 min in PBS and stained for 10 min in Hoechst (1:1000), washed 1 × 5 min in PBS and 1 × 5 min in dH2O before being mounted with Prolong Gold anti-fade reagent. Slides were allowed to dry for a while before starting microscopy.

### RNA extraction

Total RNA was extracted from both fresh tissue and culture expanded cells using a miRNeasy Mini Kit (Qiagen, Hilden, Germany). We applied some modifications to the protocol to optimize RNA extraction from fresh tissue and cells, however, the same procedures were used for cultured cells. Around 2–5 × 10^6^ of fresh cells or 1–2 × 10^6^ cultured cells were washed twice with PBS and lysed directly in 0.7 ml Qiazol (Qiagen Sciences) for 5 min at RT, then 70 *μ*l 1-Bromo-3-chloropropane (Sigma-Adrich) was added and vigorously shaken for 30 sec and centrifuged at 12 000 g for 10 min at 4 °C. After this, the aqueous phase was collected carefully and transferred to a new tube containing 0.5 ml of 100% Ethanol and mixed thoroughly. The mixture was pipetted directly onto an RNeasy Mini column and centrifuged (12000 *g*, 30 s, RT) after which the flow-through was discarded. Then 0.5 ml RPE buffer was added to the column and centrifuged (12 000 g, 30 s, RT). This step was repeated again for 1 min. 30 ul of RNase-free water was used to elute the RNA. Purity and quantity were measured by a Nanodrop spectrophotometer (Wilmington, DE, USA). For microarrays, RNA concentration and RNA quality control were determined using a 2100 Bioanalyzer. Only RNA that had a RIN value higher than 4.6 was used.

### Statistics and microarray analysis

The results of flow cytometric analysis were presented as tabular data, imported to J-Express (Molmine, Bergen, Norway)^36, 37, 38^ and analyzed using HCA (Figure 2a) and HCA with distance matrix (Figure 2b) using Pearson correlation as a distance measure and average linkage and Unweighted Pair Group Method with Arithmetic Mean (UPGMA). A non-parametric test, the Mann–Whitney test was used to estimate the significant difference in marker expression by flow cytometry, and a *P*-value <0.05 was considered significant.

For microarray, RNA was amplified and hybridized to Illumina HumanHT-12 V4.0 expression beadchip (platform GPL10558). Microarray data comply with MIAME standards and have been submitted to the Gene Expression Omnibus^39^ with GEO accession number (number currently pending). Microarray data from this microarray and the previously published microarrays with GEO accession numbers GSE41470 (Illumina, GPL10558) and GSE31262 GPL2986 (ABI Human Genome Survey Microarray Version 2, GPL2896) were imported to Microarray data analysis tool J-Express^36, 37, 38^ and quantile normalized. The fusion of the microarray data resulted in 15982 genes. For global (PCA),^40^ the data were exported from J-Express as tabular data and imported to the MultiExperiment Viewer MeV v4.8.1 (http://www.tm4.org/mev.html) for visualization. Data were log2 transformed and normalized to the mean. After filtering for the most variably expressed genes (minimum S.D.=1), 2833 genes were selected and used for hierarchical cluster analysis. Differentially expressed genes (DEGs) were identified using the grouping function and fold-change analysis in J-Express. The two groups consisted of a) brain cells isolated from SVZ and cultured as neurospheres, (serum-free and mitogen-containing medium, GSE31262) and b) UA and brain cells isolated from HPC, SVZ, GM and WM. Cells in b were cultured in 1% FBS-containing medium (GSE41470 and the current work). Using the cutoff fold-change value of ⩾3, the 2321 DEGs were selected. After filtering for most variably expressed genes (minimum S.D.=1) 1440 genes were selected and analyzed by the DAVID web-based functional annotation tool suites v 6.7 (https://david.ncifcrf.gov).^41^ Using more stringent filtering (minimum S.D.=2), 224 DEGs were selected and used for hierarchical cluster analysis. DEGs were also identified for the groups of (a) fresh cells from UA and (b) cultured cells from UA. Using the cutoff fold-change value of ⩾3, 2385 DEGs were selected. These genes were analyzed by the DAVID web-based functional annotation tool.

## Figures and Tables

**Table 1:** Cell yield obtained from ultrasonic aspiration samples of epileptic patients

**Sample**	**Total weight (g)**	**Processed (g)**	**Total cell count**	**Total viable cells**	**Viability**	**Yield (viable cells/g)**
H1255UA	N/A	N/A	276 × 10^6^	226 × 10^6^	82%	N/A
H1303UA	N/A	N/A	300 × 10^6^	170 × 10^6^	57%	N/A
H1304UA	N/A	N/A	90 × 10^6^	56 × 10^6^	62%	N/A
H1322UA	32	24	600 × 10^6^	330 × 10^6^	56%	14 × 10^6^
H1332UA	24	15	135 × 10^6^	70 × 10^6^	52%	4.7 × 10^6^
H1339UA	7	5	76 × 10^6^	33 × 10^6^	47%	6.6 × 10^6^
H1401UA	12.7	4	54 × 10^6^	20 × 10^6^	37%	5 × 10^6^
H1404UA	12.5	3	19 × 10^6^	10 × 10^6^	54%	3.4 × 10^6^
H1411UA	20	10	90 × 10^6^	58 × 10^6^	64%	5.8 × 10^6^
H1423UA	9	5.8	38 × 10^6^	32 × 10^6^	81%	5.5 × 10^6^
Mean±S.E.M.			168±56.7 × 10^6^	101±33.7 × 10^6^	59±4.4%	6.7±1.3 × 10^6^

Abbreviations: g, gram; N/A, not available; UA, ultrasonic aspiration sample
