# Supplementary material for: Identification and characterization of a new source of adult human neural progenitors
Source: Cell Death Dis. 2017 Aug 10;8(8):e2991–. doi: 10.1038/cddis.2017.368 (PMC5596556; doi:10.1038/cddis.2017.368)

Supplementary information

Inventory of supplemental information

Supplementary Table 1 (Belongs to Fig. 1)

Supplementary Table 2 (Belongs to Fig. 2)

Supplementary Fig. 1 (Belongs to Fig. 2)

Supplementary Fig. 2 (Belongs to Fig. 2)

Supplementary Table 3 (Belongs to Fig. 2)

Supplementary Fig. 3 (Belongs to Fig. 5)

Supplementary Fig. 4 (Belongs to Fig. 5)

Supplementary Fig. 5 (Belongs to Fig. 5)

Supplementary Fig. 6 (Belongs to Fig. 5)

Supplementary Fig. 7 (Belongs to Fig. 5)

Supplementary Figure legends

Supplementary Method Tables

Supplementary Figure legends

Supplementary Fig. 1 (Belongs to Fig. 2). Flow cytometry analysis showing the differences in marker expression between human dermal fibroblasts and primary cells expanded in serum-containing conditions. Fibroblasts lacked the expression of several MSC/pericytes markers including CD105, CD146 and PDGF-b, and NSC/NPC markers like SOX8, SOX9, and Nurr1, but do express very high level of CD248 and other well-known markers for MSCs/pericytes. See Table S2 for other markers. The FACS gate was set on total population after excluding cell debris and doublets using the Fluorescence Minus One Control strategy.

Supplementary Fig. 2 (Belongs to Fig. 2). Flow cytometry analysis showing the change in marker expression of cells that were cultivated in serum-containing conditions (AD10) and then transferred to neurosphere condition (AD10 to NS). The flow cytometry analysis showed that the cells downregulated and lost the marker expression of MSC (right panel), NPC and neural cells (left panel) at the same time, as well, cells seemed to die stopped proliferating as indicated by Ki67 staining (end of right panel).

Supplementary Fig.3 (Belongs to Fig. 5). Principal component analysis 2D plots.

a) PCA1 vs PCA2, b) PCA1 vs PCA3 and c) PCA2 vs PCA3.

Supplementary File 1: Functional annotation analysis showing genes 2321 DEGs, the most variable DEGs (1440) and the most variable DEGs under especially stringent filtering (224) and pathways that are differentially expressed in spheres and adherent cells. See Methods for details on bioinformatics filtering.

Supplementary Fig. 4 (Belongs to Fig. 5). HCA of the 3883 genes showing the most variable genes after filtering. Included in this analysis are: a) the new samples from this study (GEO set number pending), and the samples from our previous analyses (Murrell et al., 2013 and Sandberg et al., 2013). The dendogram colors red, blue and green specify mesenchymal, neural/proneural and classical subtypes, respectively. The expression values were $\log_2$ transformed.

Supplementary Fig. 5(Belongs to Fig. 5). HCA of the 1440 genes showing the most variable genes after filtering of 2321 DEG differentially regulated between Sp and AD.

Supplementary Fig. 6 (Belongs to Fig. 5). ERK pathway

Supplementary Fig. 7 (Belongs to Fig. 5). HCA of the 2385 genes showing genes that were differentially expressed between the groups of fresh samples and cultured cells.

Supplementary Method Tables . Antibodies used for flow cytometry and Immunocytochemistry

Directly conjugated antibodies	Producer	Primary Antibodies	Producer
CD133/2- APC	Miltenyi	Rabbit anti-alpha smooth muscle Actin (αSMA)	Abcam
CD133/2- PE	Miltenyi	Goat anti-Chitinase 3-like 1 (YKL40)	R & D Systems
CD56-FITC	Miltenyi	Rabbit anti-SOX9 (clone EPR12755)	Abcam
CD44-APC	eBioscinece	Rabbit anti-SOX8 (H-95)	Santa Cruz
CD15-APC	Miltenyi	Mouse anti-SOX5	Abcam
CD15-PE	Miltenyi	Mouse anti-SOX2 (monoclonal antibody)	Cell Signaling
CD34-APC	BD Pharmingen	Goat anti-Vimentin	R & D Systems

CD146-APC	Miltenyi	Mouse anti-MAP2A	Millipore
CD31-FITC	Miltenyi	Mouse anti-Endosialin, clone B1/35	Millipore
CD271-APC	Miltenyi	Rabbit anti-CD271	Abcam
CD166-PE	BD Pharmingen	Mouse anti-O4, clone 81	Chemicon
CD9-FITC	eBioscinecе	Mouse anti-hNestin	Abcam
CD9-PE	eBioscinece	Rabbit anti-Ki67 (Clone SP6)	Abcam
Nestin-AF647	BD Pharmingen	Rabbit anti-PDGFB	Sigma Aldrich
GFAP-FITC	BD Pharmingen	Mouse anti-PDGFR-B	Sigma Aldrich
CXCR4-PE	Miltenyi	Rabbit anti-PDGFR-α (D13C6)	Cell Signaling
CD59-FITC	Biolegend	Doublecortin (c-18)	Santa Cruz
		Mouse anti-S100 Antibody [8B10]	Abcam
		Rabbit anti-c-Kit	Abcam
		Rabbit anti-GFAP	Dako
		Mouse anti-NeuN	Millipore
		Mouse anti-CD106	eBioscinece
		Rabbit anti-EGFR (D38B1)	Cell Signaling

Secondary Antibodies used for FACS and Immunocytochemistry.

**Secondary Antibodies**	**Producer**
Alexa Fluor 488 donkey anti-rabbit	Molecular Probes
Alexa Fluor 488 donkey anti-mouse	Molecular probes
Alexa Fluor 647 donkey anti-goat	Molecular probes
Alexa Fluor 647 donkey anti-Rabbit	Molecular probes

Alexa 647 donkey anti-mouse	Life Technologies
Alexa 594 Donkey anti-Goat	Molecular probes
Alexa 594 donkey anti-rabbit	Life Technologies

Supplementary Table 1. Patient diagnosis, age and gender of samples included in this study.

Sample	Diagnosis	Age	Gender
**H1255**	Hippocampus sclerosis	51	Female
**H1303**	Cortical dysplasia	1	Male
**H1304**	Normal temporal lobe	24	Female
**H1317**	Hippocampus sclerosis	44	Female
**H1339**	Hippocampus sclerosis	33	Female
**H1401**	Cortical dysplasia	45	Female
**H1404**	Hippocampus sclerosis	44	Male
**H1411**	Hippocampus sclerosis	42	Female
**H1423**	Normal hippocampus	43	Female
**H1433**	Hippocampus sclerosis	26	Female
**H1434**	Normal temporal lobe	23	Female
**H1446**	Hippocampus sclerosis	29	Female
**H1702**	Hippocampus sclerosis	22	Male
**H1703**	Cortical dysplasia	49	Female

Supplementary Fig. 1

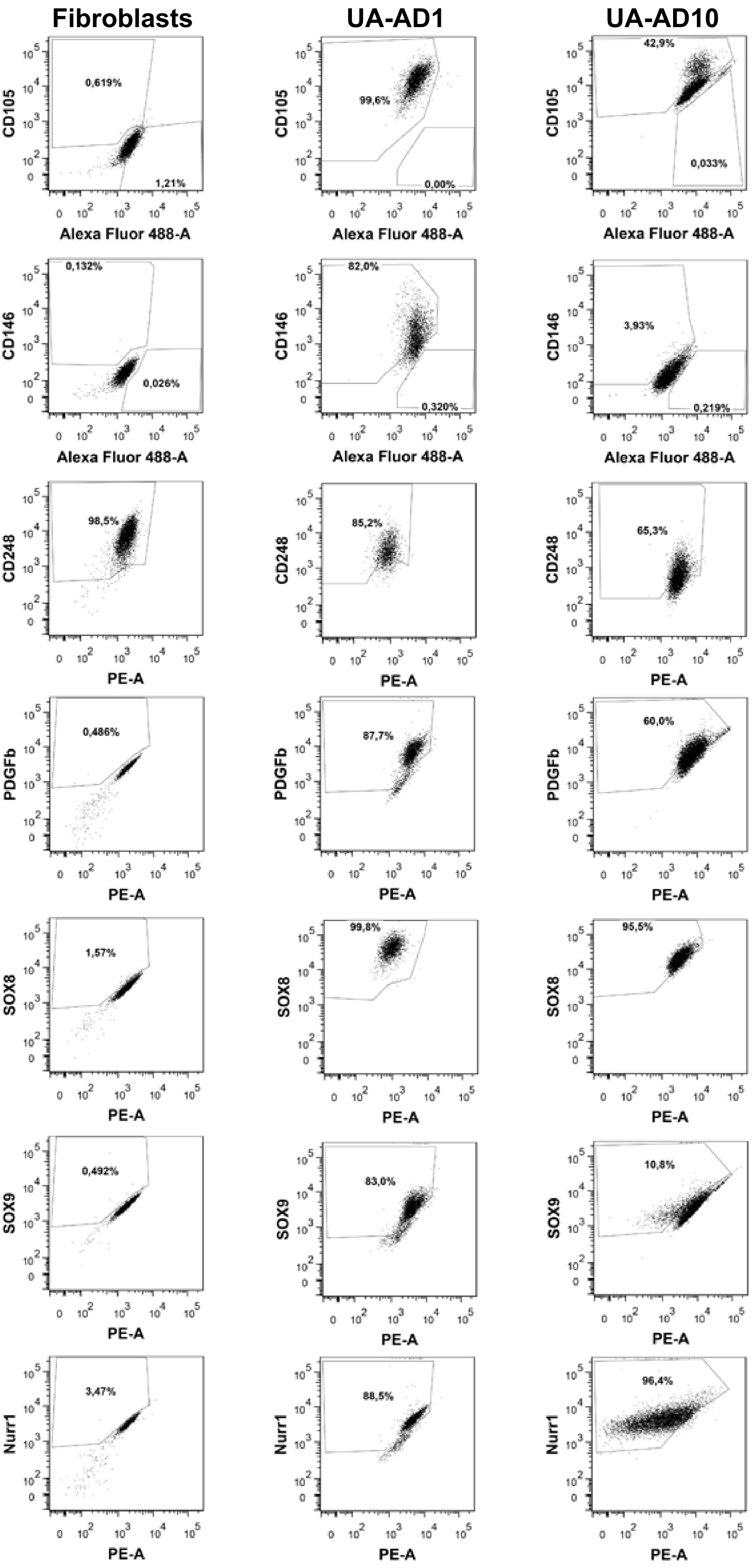

Supplementary Fig. 2

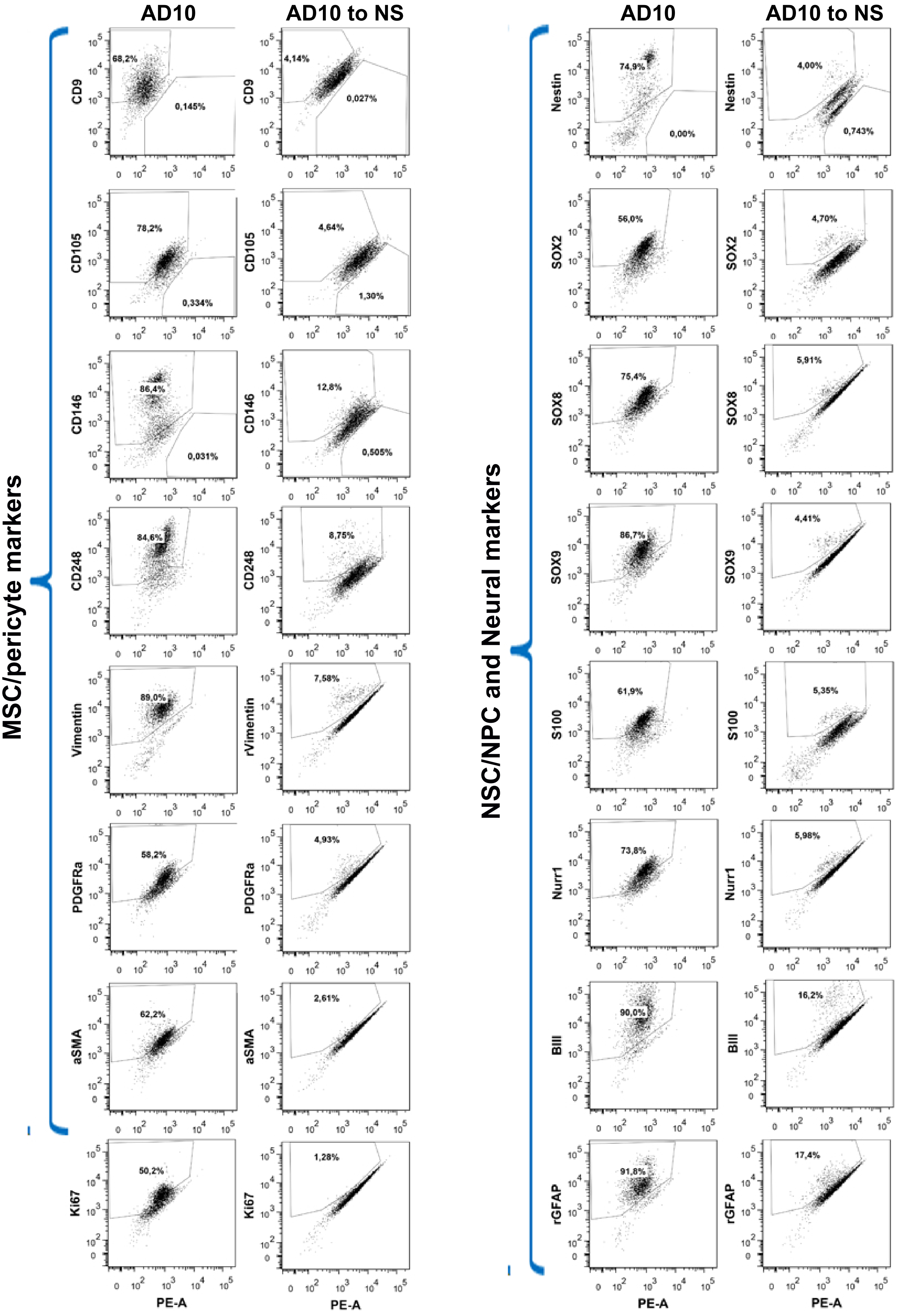

Supplementary Fig. 3

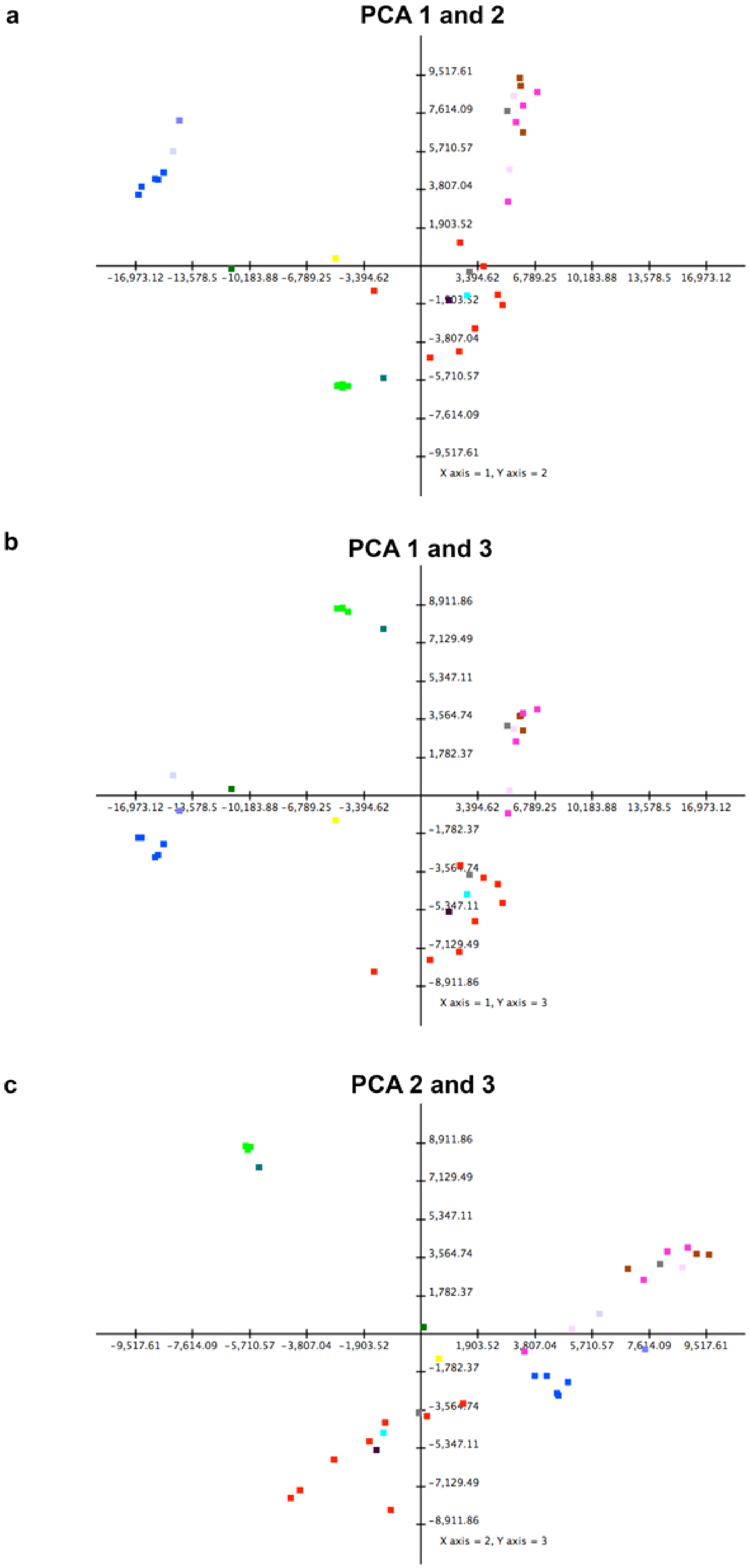

Supplementary Fig. 4

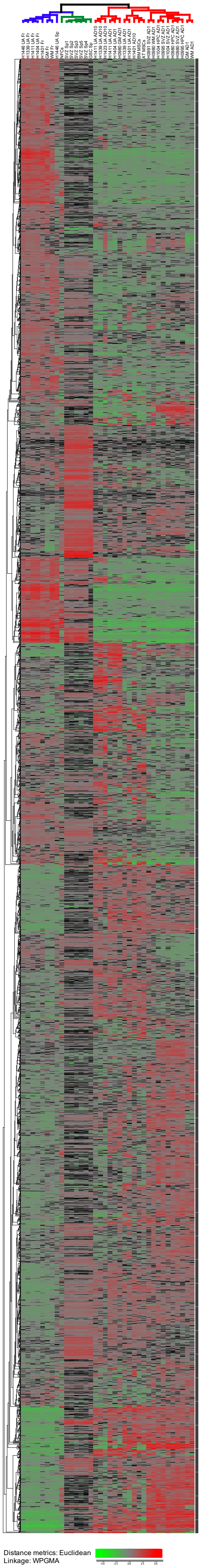

Supplementary Fig. 5

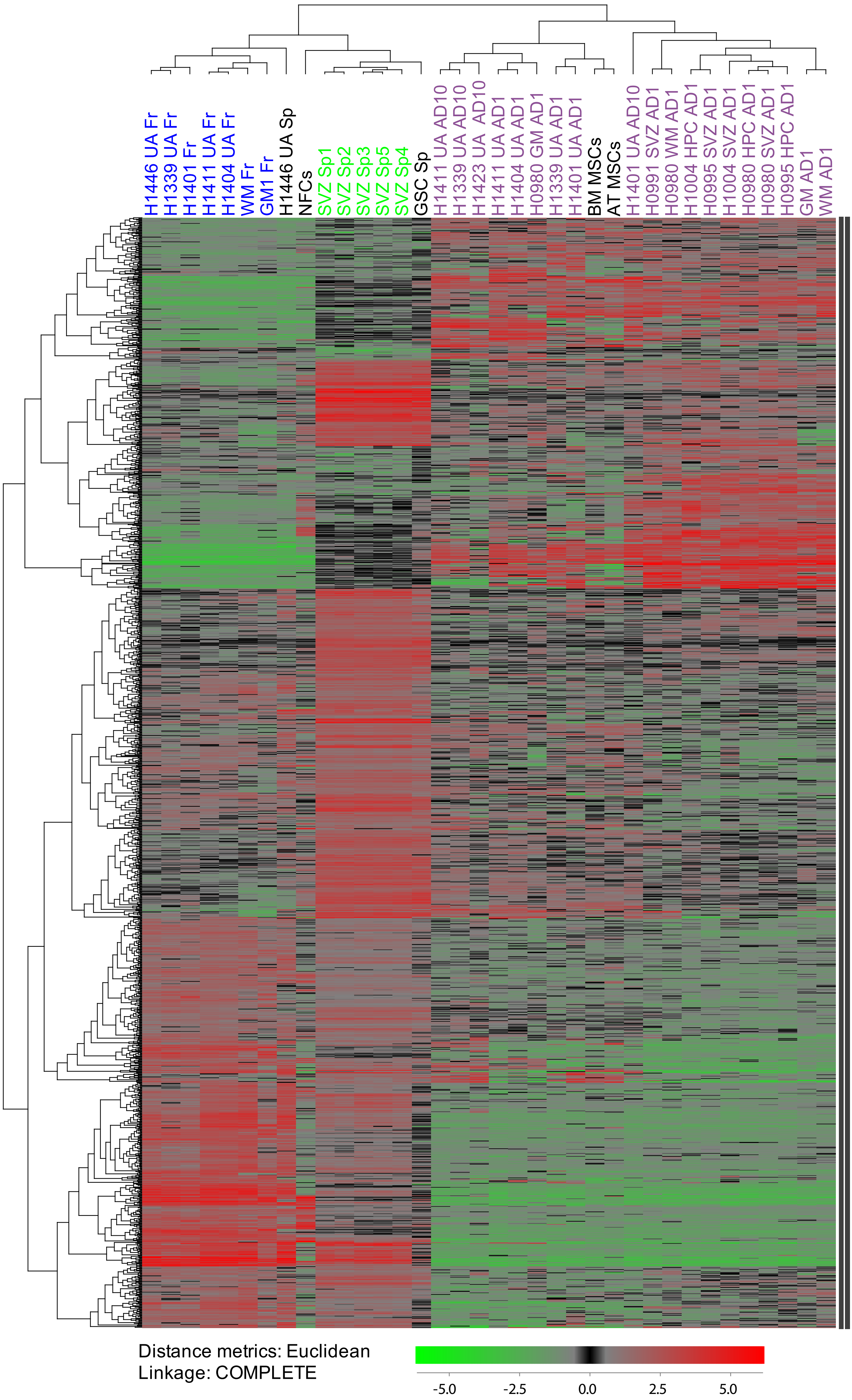

Supplementary Fig. 6

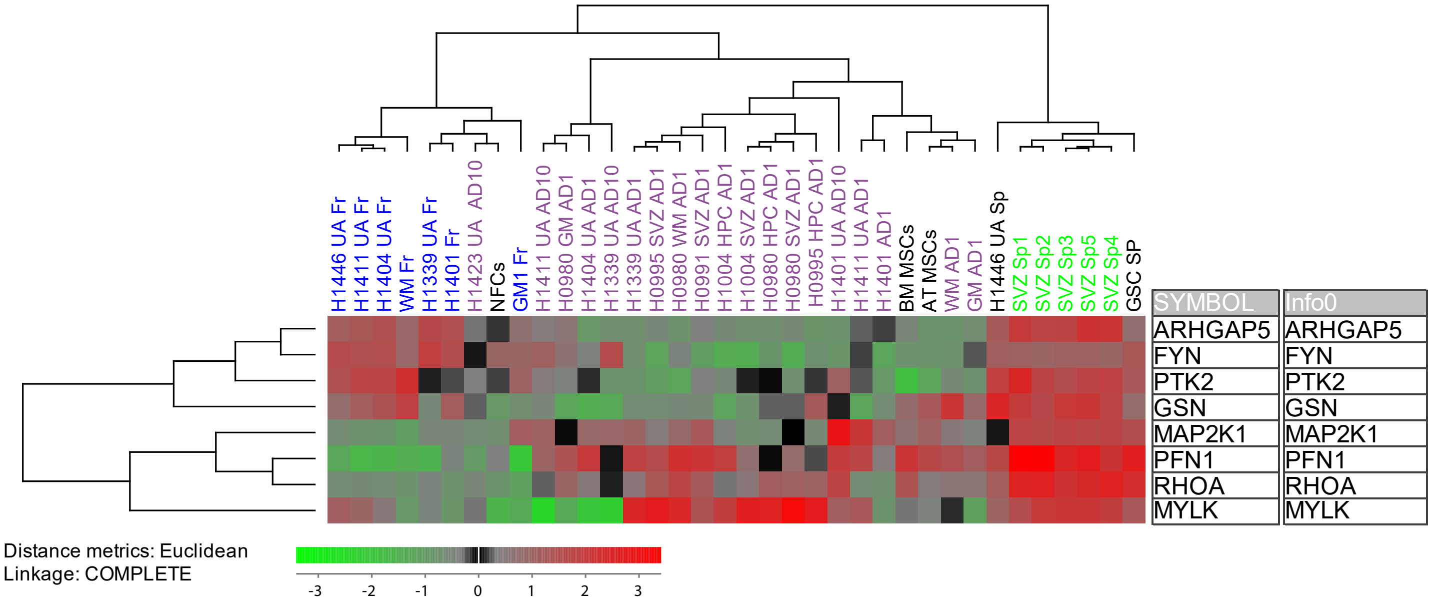

Supplementary Fig. 7

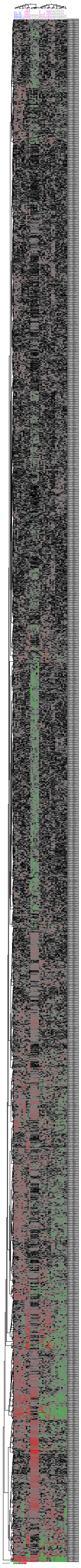

Supplement: Supplementary Information [file cddis2017368x1.pdf]
